# What do health insurance deductibles do to health care spending growth?

**DOI:** 10.1007/s10754-026-09416-y

**Published:** 2026-05-26

**Authors:** Claudio Lucarelli, Molly Frean, Aliza S. Gordon, Lynn M. Hua, Mark V. Pauly

**Affiliations:** 1 The Wharton School, University of Pennsylvania, 3641 Locust Walk, Philadelphia, PA 19104 USA; 2 Analysis Group, Boston, MA USA; 3 Elevance Health Public Policy Institute, Washington, DC USA; 4 Robinson College of Business, Georgia State University, Atlanta, GA USA

**Keywords:** Health insurance, High deductible, Spending growth, Insurance plans, I13

## Abstract

Costly new technology, while often beneficial, has been identified as one of the principal drivers of healthcare spending *growth*. Recent literature has shown high deductible health plans (HDHPs) can have an immediate negative impact on *levels* of healthcare spending, but their effects on spending *growth* remain unknown. Analyzing a panel of multiple-employer-group claims data from a national insurer that is extensive enough to identify long run effects on spending *growth*, we find that enrollment in HDHPs for four or more years is not associated with lower total health care spending *growth *(or lower *growth *in medical services spending) compared to persistent enrollment in low deductible health plans (LDHP).

## Introduction

Health care spending per capita for the privately insured population in the United States has grown rapidly for many decades, exceeding *growth *in wages, consumer prices, and GDP. There has been ongoing interest in how insurance coverage can be reformed to reduce this *growth*, ideally limiting it to an amount commensurate with national income and improvements in quality and health outcomes. As noted by Brot-Goldberg et al. (2017), a necessary piece of evidence for that change in spending *growth *is understanding how consumers respond to cost-sharing.

However, while their study is one of a number showing that transitioning a population of insureds from low to high deductible plans is associated with a significant reduction in spending soon after the transition, there is no evidence on how higher cost-sharing affects spending *growth *(if at all) for years following. Managed care, changes in provider payment methods, and increasing patient cost-sharing have all been shown to have some effect on lowering the *level* of spending as they are phased in, but, after they have been adopted and diffused, there is strong evidence that spending *growth *tends to resume its former path (Chernew & Newhouse, 2011; Cutler et al., 2000; McWilliams et al., 2016; Manning et al., 1987). Does spending *growth *after increases in cost-sharing associated with high deductible plans follow the same pattern of returning to prior magnitudes as we have witnessed with other interventions?

Why might *growth*, after a dip, return close to its former rate? The primary reason for spending *growth*, and the one that motivates this study, is that it is thought to be largely driven by continuous incremental spending on better but more costly new technology (Smith et al., 2009). Chernew and Newhouse (2011) made the point that changes in *levels *of cost-sharing should only affect spending *growth *on existing technologies (by reducing moral hazard) as higher cost-sharing is being phased in. After that, any effects of cost sharing on *growth *will depend on the responsiveness of demand for new technologies.

That is, higher cost-sharing has the potential to reduce spending *growth* – and not just spending *levels* – if it limits the adoption and diffusion of new and costly (but potentially beneficial) technologies (Chandra & Skinner, 2012; Weisbrod, 1991). Paradoxically, however, there has been very little investigation of whether and how the level of cost-sharing affects the *growth *of health care spending. Early work by Peden and Freeland (1998) using national health expenditure data found a (time series) estimate of a significant impact of the proportion paid out-of-pocket on the *growth *of spending, but no evidence of an impact of changes in this share on contemporaneous changes in spending. Golberstein et al. (2013) examined the impact of private Medigap insurance on the level and *growth *of Medicare spending and found that supplemental coverage raised both. However, the effect of cost-sharing on privately insured spending *growth *has been understudied, despite serious policy concern about spending *growth *and the commonly hypothesized potential for increasingly prevalent high deductible plans to curb it.

High deductible health plans (HDHPs) have become increasingly popular in the employment-based market and a key object of policy interest. Since their introduction to the market, HDHPs have been promoted to reduce healthcare spending. “Bronze” plans on the Affordable Care Act’s individual insurance exchanges are characterized by high deductibles and are the second-most popular type of plan. In a review of the effects of consumer-directed health plans (CDHPs), the term given to HDHPs coupled with tax-favored health savings accounts (HSAs), healthcare expenditures were reported to have decreased between 5 and 14%, depending on the study, with greater reductions in healthcare spending for plans with larger deductibles (Bundorf, 2016). Empirical evidence has demonstrated that HDHPs may lead to significant spending reductions in the short term as consumers are switched to greater exposure to the cost of current types of care. However, the potential impact on spending *growth *for insureds who remain in HDHPs long after any initial impact is unknown. We, therefore, seek to answer the question: compared to LDHPs, do HDHPs in fact encourage less use of the new but more expensive types of care that arrive every year?

There are some patterns in the differential impact of HDHPs on the *levels* of use of specific health care services. Past research has documented that when CDHPs reduce healthcare spending, a portion is usually driven by reductions in outpatient care and pharmaceutical spending, without a clear effect on inpatient admissions (Buntin et al., 2011; Charlton et al., 2011; Lo Sasso et al., 2010a, b; Parente et al., 2008). There have been exceptions, such as one study where enrollment in an HDHP increased the utilization of drugs and had no effect on outpatient care relative to a PPO plan (Waters et al., 2011). The presence of high deductibles seems even to deter the use of care not subject to the deductible, such as some covered preventive services, though that effect may come from an impact on complementary physician services.

There have also been mixed results on the differential impacts of cost-sharing on the quality of care, typically measured by the appropriateness of services. For example, in the RAND Health Insurance Experiment (Manning et al., 1987; Newhouse et al., 1993), higher cost-sharing reduced care labeled both clinically necessary and unnecessary (though often with no effect on health outcomes). Some more recent studies (Wharam et al., 2007, 2011) suggest that consumers can differentiate between more or less clinically appropriate care, while others found that, with less insurance coverage, they nevertheless reduced the use of recommended preventive care (Buntin et al., 2011) and reduced the cervical and breast cancer screening rates (Charlton et al., 2011; Wharam et al., 2018). There have also been mixed results on the differential impacts of HDHPs on vulnerable, disadvantaged populations such as those who are low-income and high risk (Davis, 2004; Eisenberg et al., 2020; Gaffney et al., 2020; Haviland et al., 2011; Rabin et al., 2020; Woolhandler & Himmelstein, 2007).

In this study we use a novel data set to explore the path of health care spending following a change from a low deductible plan to a high deductible plan and then remaining in the high deductible plan. We study a population that remained in high (or low) deductible plans for a minimum of four years—and with many in such plans for longer because (given the relatively low turnover rates we observed) they had transitioned to HDHP before the beginning of the observation period. We also use a different sample of switchers from the data set to confirm the finding of others that individuals who switch into HDHPs from LDHPs experienced a significant drop in spending immediately after the switch. However, we then find that subsequent *growth *in medical spending is not significantly different for those who persisted in HDHPs compared to those who persisted in LDHPs*.* Enrollment in HDHPs may therefore not reduce access to new medical technologies and offering special tax advantages to high deductible plans may not be an effective cost containment device.

Two studies related to but different from our work are by Brot-Goldberg et al. (2017) and Haviland et al. (2016). Brot-Goldberg et al. (2017) study a natural experiment where employees of a single large firm were mandated to switch from a generous zero-deductible plan that provided free in-network health care to a HDHP paired with a subsidized HSA. Access to the same providers remained post-switch to the HDHP plan. They estimate that the switch caused a spending reduction of approximately 12% of firm-wide spending using four years of pre-switch and two years of post-switch data in the time window 2006—2015. When decomposing this effect, they find no evidence of increased price shopping, but rather that consumers reduced quantities of both potentially low-value and higher-value care (though somewhat more for presumably low-value imaging). Their results are consistent with greater sensitivity to spot prices, incurred at the point of service, over true shadow prices, which take into consideration expected spending over the entire plan benefit year. However, because their study was limited to only two years of data from workers who transitioned from low to high deductible insurance in a single (large) firm, their empirical study (and a number of similar “switching” studies) could only investigate the impact of coverage differences on the level of spending and not on the subsequent rate of *growth *of spending. We find similar reductions in spending in the short run from our analysis of a sample of enrollees that switched from LDHP to HDHP.

In a large multi-employer study, Haviland et al. (2016) use a difference-in-differences approach to quantify an intent-to-treat effect of employer CDHP offer on spending *growth *for up to three years. These authors find that in each of the three years after firms offer a CDHP, there was a 5% reduction in total health care spending relative to a baseline of firms only offering LDHPs. They find that decreases in total spending are driven primarily by reductions in spending on outpatient care and prescription pharmaceuticals; by the third year, there are no differences in spending for inpatient and emergency department care. In our analysis of subjects with longer tenures in each plan type, by contrast, we reach a different conclusion about the long run: the *growth *in healthcare spending after the transition period for those individuals in HDHPs follows the same trajectory as those in LDHPs, and therefore, HDHPs do not lower the *growth *in spending as Haviland et al. (2016) might have suggested.

A key limitation of the prior literature is that the persistence of the impact of HDHPs on healthcare spending, specifically whether they lower *growth *over time, remains unclear. Our study provides the first large-sample investigation of the relationship between private insurance deductibles and the *growth *of privately insured spending over a long period of time. We build upon past research and examine the effect of HDHPs on the *growth *of health care spending using a large national sample of claims and enrollment data from Elevance Health, a private national insurer. Our data includes 53,721 employer groups across 14 states over the four-year time period from 2015 to 2018 and allows us to describe and analyze patterns of spending *growth *for different types of covered services for populations that have remained in HDHP or LDHP plans for at least four years and longer if the transition to HDHP occurred prior to our study period.

Our empirical approach examines how HDHPs affect spending *growth* driven by the ongoing diffusion of medical technologies across the health care system during 2015—2018. We do not limit our analysis to only technologies introduced during this specific period; rather, we observe how plan generosity affects responses to the continuous process of technology adoption and diffusion—including both new innovations and the spread of previously established technologies.

We implement the Callaway and Sant'Anna (2021) difference-in-differences estimator, which is specifically designed for staggered adoption settings and uses doubly-robust estimation to properly account for differential timing of treatment and heterogeneous treatment effects over time. We initially focus on a large sample of members who were consistently insured with either high- or low-deductible coverage over the study period (“stayers”). Our results for this sample indicate that high deductibles are associated with mixed effects on spending *growth*. Relative to members enrolled in LDHPs, members enrolled in HDHPs do not experience lower dollar *growth *in total spending (and associated insurance claims), but they do have lower *growth *in prescription drug spending. Because individuals are not randomly assigned to a particular insurance plan, we exploit the variation in the employers’ choice sets to alleviate concerns about selection. Some employers in our sample exclusively offer a HDHP or LDHP, whereas others offer both high and low deductible options. We exploit this variation across employers, and also within employers over time to understand the effect of HDHPs on spending *levels *and *growth *conditional on the ability to choose. Accounting for selection bias this way, we confirm that HDHPs affect spending *levels *but have no impact on total spending *growth *in our sample. We also find that adverse selection manifests itself on the *levels *of spending but not on its *growth*.

To further explore selection bias, we analyze a sample of members who were forced to switch from LDHP to HDHP coverage during the study period. We find that such members experience lower spending relative to members continuously enrolled in either plan type around the time of the switch consistent with the findings of Brot-Goldberg et al. (2017), however, this reduction does not persist. This result is robust to controlling for unobserved and permanent risk factors that could drive selection when accounting for within-employer variation in deductible choice over time.

The overall pattern in our data therefore is one of no HDHP effect relative to LDHP on aggregate spending *growth *for all insured care. It appears that HDHPs do not have as much potential for long-term cost containment as their advocates contend. However, this work compares spending *growth *between members of different plans across the country from a single insurer. We are not able to observe plans from other insurers or examine employer-level selection with the full plan menus offered. While we can observe employer group identifiers and track plan offerings over time, we do not have detailed employer characteristics such as industry sector or firm size beyond the employer group classification. This means that we cannot identify the impact of high deductibles on overall spending in markets where HDHPs offered by many insurers have had substantial penetration compared to markets where such plans are rare. As Finkelstein (2007) has noted, effects of cost-sharing estimated at the individual insurance plan level may well understate those at the market level.

The next section offers a simple model of plan choice and spending conditional on plan, which helps to identify the main sources of selection and motivates our empirical analysis. Section "Data" describes the data, Section "Estimation" describes our estimation strategy, Section "Results" presents our results, and Section "Conclusion" concludes.

## Model

In this section, we develop a two-stage individual level model that first describes the considerations behind a decision to enroll in either a HDHP or a LDHP (when such a choice exists in employer-sponsored coverage), and then describes the choice of medical care expenditure conditional on insurance choice.^1^ Our concept of the underlying mechanism is that new technology is the main driver of spending *growth *and old technology impacts mostly *levels *of spending. If the demand for new technology is more inelastic to changes in coverage, the introduction of HDHP is hypothesized to have less of an impact on the utilization of new technology and therefore on spending *growth*, and a larger impact on old technology and therefore on *levels *of spending (Weisbrod, 1991). The model allows for the choice of technologies of different marginal health product, which provide insights about consumers’ response to cost sharing for low value and high value care. Consistent with individuals’ dynamic optimization behavior within the insurance year, our exposition and solution of the model uses backward induction. Although most of the employers in our sample offer only one level of deductible in the plan administered by the insurer, according to our data about 35% of employers offered insurer-administered plans with different deductibles.

Conditional on the choice of insurance plan deductible, the insured person is assumed to choose medical care expenditure according to a simplified version of the Grossman (1972) model, where medical care expenditure is an input for the production of health. We consider expenditure on two medical inputs, x_1_ and x_2,_ which differ on their health production ability (their marginal health product) and are translated into a utility flow of health through the function $f\left(.,.\right),$ which is assumed to be twice continuously differentiable and exhibit decreasing marginal returns. The insured’s objective function depends on health and the residual consumption after paying for insurance premiums and medical care, with the function g(.) transforming dollars to utility units. We simplify the problem by summarizing the degree of coverage with the parameter $\upalpha$, where higher values of $\upalpha$ can be interpreted as coverage with lower deductibles.^2^ The maximization problem is as follows, where *W* represents income and π the insurance premium:

$$\underset{{x}_{1},{x}_{2}}{Max} f\left({x}_{1},{x}_{2}\right)+g\left(W-\uppi -\left(1-\upalpha \right)\left({x}_{1}+{x}_{2}\right)\right)$$

The first order conditions are:

$$\begin{array}{c}{f}_{{x}_{1}}\left({x}_{1},{x}_{2}\right)-{g}_{{x}_{1}}\left(1-\upalpha \right)=0\\ {f}_{{x}_{2}}\left({x}_{1},{x}_{2}\right)-{g}_{{x}_{2}}\left(1-\upalpha \right)=0\end{array}$$

Both conditions show that with no coverage $(\upalpha =0$), marginal benefits as measured by marginal health product equal marginal costs. However, the presence of insurance ($\upalpha>0$) distorts the choice of medical care expenditure, generating the problem of moral hazard.

To understand how the choice of expenditure on medical inputs varies with coverage, we totally differentiate the first order conditions to obtain:

$$\left[\underset{< 0}{\underbrace{{f}_{{x}_{1}{x}_{1}}+\left(1-\upalpha \right){g}_{{x}_{1}{x}_{1}}}}\right]d{x}_{1}-\left[\underset{< 0}{\underbrace{\left(1-\upalpha \right){g}_{{x}_{1}{x}_{1}}-{g}_{{x}_{1}}}}\right]d\upalpha = 0$$

Above, both terms in brackets are negative due to the decreasing marginal return assumptions ($${f}_{{x}_{1}{x}_{1}}<0$$,$${g}_{{x}_{1}{x}_{1}}<0$$), and increasing utility on consumption ($${g}_{{x}_{1}}>0)$$. The same applies for expenditure on x_2_.

Therefore, expenditure on medical care is increasing in coverage as is shown in the following equations:

$$\begin{array}{c}\frac{d{x}_{1}}{d\upalpha } = \frac{\left(1-\upalpha \right){g}_{{x}_{1}{x}_{1}}-{g}_{{x}_{1}}}{{f}_{{x}_{1}{x}_{1}} + \left(1-\upalpha \right){g}_{{x}_{1}{x}_{1}}}> 0\\ \frac{d{x}_{2}}{d\upalpha } = \frac{\left(1-\upalpha \right){g}_{{x}_{2}{x}_{2}}-{g}_{{x}_{2}}}{{f}_{{x}_{2}{x}_{2}} + \left(1-\upalpha \right){g}_{{x}_{2}{x}_{2}}}> 0\end{array}$$

Given these observations, we obtain for each individual a demand curve for medical care – derived largely, as Grossman (1972) suggests, from the demand for health – which will depend on income, insurance coverage as measured by marginal user price, preferences for health versus other goods, market prices for health care goods and services, time cost associated with consuming health care goods and services and other desired consumption items, and provider advice.^3^ All of the arguments except the last one would be present in any economic demand function where there is insurance that takes the form of health insurance. This demand function would be similar to others for a consumer’s decision to initiate contact with the health care system (where there is no prior physician information).

As we stated above, the inputs to the production of health x_1_ and x_2_ differ in their marginal health product, which represents the difference in value to the consumer. Suppose the schedule of diminishing marginal product for x_2_ is always above that for x_1,_ in the following way: ${f}_{x2}\left({x}_{1},{x}_{2}\right){=\upbeta f}_{{x}_{1}}\left({x}_{1},{x}_{2}\right)$ for $\upbeta> 1$. In other words, x_2_ exhibits larger marginal health product relative to x_1_. If there are no differences in the marginal (dis)utility of a dollar spent on either input (i.e., if the individual does not derive utility for the identity of the inputs beyond their contribution to health), then the derivatives of the function g(.) are identical for both inputs. Therefore,

$$\frac{d{x}_{2}}{d\upalpha } = \frac{\left(1-\upalpha \right){g}_{{x}_{2}{x}_{2}}-{g}_{{x}_{2}}}{{\upbeta f}_{{x}_{1}{x}_{1}} + \left(1-\upalpha \right){g}_{{x}_{2}{x}_{2}}} \Rightarrow \frac{d{x}_{2}}{d\upalpha } < \frac{d{x}_{1}}{d\upalpha }$$

The expression above states that the coverage elasticity of expenditure on the more productive input x_2_ is more inelastic than for the less productive input x_1_. This implication of our model emphasizes different magnitudes of response to changes in coverage (e.g., through changes in deductibles) for high value vs. low value care, or old vs. new technology, which in turn, can generate different magnitudes of change in spending *levels *or spending *growth*. To the extent new technology is more productive than older technology, this result suggests that responsiveness to increased cost-sharing will be smaller for spending *growth *associated with new technology than for spending *levels *associated with older technology.

In the first stage of the model, the individual decides to enroll in either type of plan, high or low deductible or in the simple case in which an employee is offered only one coverage option, we assume the employee is forced to take it. We do not consider the possibility of someone choosing an outside option or to be uninsured if offered insurance. The expected utility of a worker holding an insurance policy with a deductible is given by Eq. (1), where a contract is described by the premium $\pi$, the deductible D (which could be low or high), and a cost sharing rate *c*. The expected utility maximizer faces a probability *p* of experiencing a loss in wealth equal to *L*.

1 $$U\left(\pi , D, c\right) = \left(1-p\right) U\left(W - \pi \right) + p U\left(W - \pi - D-c(L-D)\right)$$

To simplify the problem further and to emphasize the role of the deductible, we initially assume the coverage after the deductible is the same for both plans, and it is very generous. A worker facing the choice of HDHP or LDHP will be indifferent between the two choices if:

$$U\left({\pi }^{H},{D}^{H}\right)=U\left({\pi }^{L},{D}^{L}\right)$$

which by the definition of expected utility provided in (1) becomes:

2 $$\left(1-p\right) U\left(W-{\pi }^{H}\right)+pU\left(W-{\pi }^{H}-{D}^{H}\right)=\left(1-p\right) U\left(W-{\pi }^{L}\right)+p\left(W-{\pi }^{L}-{D}^{L}\right)$$

Rearranging the above equation and collecting terms, we get the following expression:

$$p\left[U\left(W-{\pi }^{H}-{D}^{H}\right)-U\left(W-{\pi }^{H}\right)-\left(U\left(W-{\pi }^{L}-{D}^{L}\right)-U\left(W-{\pi }^{L}\right)\right)\right]=U\left(W-{\uppi }^{L}\right)-U\left(W-{\uppi }^{H}\right)$$

Performing a Taylor expansion around W, the utilities above can be approximated by:

$$U(W - A) \approx U(W) - A u^{\prime}(W) + \frac{1}{2} {A}^{2}u^{\prime\prime}(W)$$

Which, after some algebra, can be written as:

3 $$p[({D}^{H} - {D}^{L}) U^{\prime}(W)] \approx - U^{\prime}(W) ({\pi }^{L} - {\pi }^{H}) + U"(W)\frac{({\pi }^{L}- {\pi }^{H}) ({\pi }^{L}+{\pi }^{H})}{2}$$

Defining $\overline{\uppi }=\frac{{\uppi }^{L}+{\uppi }^{H}}{2}$, Eq. (3) can be expressed as a compact expression that describes the indifference between an HDHP and a LDHP as a function of the differences in premia and deductibles, the probability of a loss, and the coefficient of risk aversion. This expression resembles analogous expressions derived by Chiappori and Salanie (2000) and Cohen and Einav (2007) in the context of car insurance. Less risk averse individuals and those with a lower probability of experiencing a loss (which could be interpreted either as pure selection or selection on moral hazard) will prefer HDHPs.

$$\frac{\frac{p\left(D^H-D^L\right)}{\pi^L-\pi^H}-1}{\overline\pi}\approx\frac{-U^{\prime\prime}(W)}{U^{\prime}\left(W\right)}$$

The above expression captures the sources of selection bias, which are addressed in our empirical analysis, and motivate our identification strategy. Exploiting individuals who are forced to switch plan types allows us to control for unobserved risk aversion, which is assumed constant within individuals. By exploiting variation in deductible choice both across workers and over time, we are able to study situations in which there is a single choice of premium and deductible.

All else equal, higher patient cost-sharing will reduce moral hazard, which in turn will offer stronger incentives to reduce the use of beneficial medical care with low marginal benefit relative to its cost or price, as is shown in the first order conditions of our model as $\alpha$ decreases. It will also offer incentives to search more aggressively for lower unit prices if they vary in the market, if expected annual spending falls between the low and high deductible, and if the cost (and effectiveness) of search is moderate. The data we use are from a population and time period characterized by little change in overall health risk and demographic characteristics. As a result, we abstract from any effect of changes in health or in the demand for health in our analysis.

The role of exogenous price changes in our analysis is more complex. There is no a priori reason to imagine that the change in average market prices for medical services received by providers should be related to an individual’s type of insurance coverage; it should be experienced similarly by all people regardless of the type of coverage. However, because health care markets are not perfectly competitive, there is usually a range of prices a buyer could face in a market, and it has been suggested that higher cost-sharing may prompt more intensive search for lower prices. Brot-Goldberg et al. (2017) found no evidence of such shopping behavior in a static setting. However, it is plausible that insureds with higher deductibles will have more of an incentive to search for lower prices if overall prices in their area increase but the distribution of selling prices from which they must search remains the same. Hence, an effect of deductible *levels *on spending *growth *may reflect a combination of both changes in quantities and insurance-influenced changes in prices. As our data do not permit precise measurement of unit price changes, especially for new technology, we do not separate spending *growth *into these two components.

These theoretical predictions have direct empirical implications. Our model shows that if new technology has higher marginal health product than existing technology, then increased cost-sharing from HDHPs should have a larger effect on spending *levels *(driven by existing, lower-value technologies) than on spending *growth *(driven by new, higher-value technologies). We test this prediction by examining how forced switches from LDHPs to HDHPs affect spending in a period characterized by ongoing technology diffusion. Our study period captures not just the introduction of new technologies, but their continued adoption and spread throughout the healthcare system. Finding a one-time reduction in spending *levels *with no persistent effect on *growth *would be consistent with new technology having higher marginal product than established care.

## Data

We use data from the Healthcare Integrated Research Database (HIRD^SM^), a large administrative claims database containing medical and pharmacy claims for members in commercial health plans administered by Elevance Health, Inc. Elevance Health is the country’s second largest private insurer, with employer plans offered in 14 US states: California, Colorado, Connecticut, Georgia, Indiana, Kentucky, Maine, Missouri, Nevada, New Hampshire, New York, Ohio, Virginia, and Wisconsin.

Our dataset includes 53,721 employer groups observed between 2015 and 2018, consisting of 46,612 small group employers (87%) and 7,109 large group employers (13%). We observe employer group identifiers that allow us to track plan offerings over time and identify changes in available plan options. However, we do not have detailed employer characteristics such as industry sector or specific firm identifiers beyond the employer group classification.

The claims data contain fields common to most administrative claims datasets, including diagnosis and procedure codes (medical claims) and national drug codes (NDCs; pharmacy claims). Cost data contained in the claims include member out-of-pocket payments (from deductibles or coinsurance/copayments), payments by the plan and total allowed amounts. Unless otherwise indicated, results presented in this paper regarding spending are based on total allowed amounts and therefore include payments made by both members and Elevance Health. We separately analyze spending for medical care and prescription drugs. Medical spending includes spending in the inpatient, outpatient, emergency room and skilled nursing facility settings, while drug spending includes outpatient prescription drugs.

The study sample includes adult members aged 18—64 enrolled in Elevance Health plans designated as preferred provider organization (PPO) products through a sponsoring employer. All plans are fully-insured by Elevance Health; our sample does not include plans administered by Elevance Health on behalf of employers who choose to self-insure. Each member had continuous medical and pharmacy coverage from 2015 to2018 (the study period). During this period, the U.S. health care system experienced ongoing technology diffusion including the continued adoption of cancer immunotherapy checkpoint inhibitors, expansion of biosimilars and specialty pharmaceuticals, and advances in diagnostic imaging. Our analysis captures how HDHPs versus LDHPs affect spending *growth *driven by this continuous diffusion process, rather than focusing solely on the initial introduction of new technologies. While Elevance Health also offers insurance products on the non-group (individual) market, such members were excluded in an effort to reduce selection bias. Precise plan benefit design data including the plan deductibles are directly observed — available from Elevance Health but separate from the HIRD^SM^ — were used to classify plans according to the level of their individual and/or family deductibles.^4^

We defined HDHPs as plans with an individual deductible of at least $1,250 or a family deductible of $2,500. If a member’s plan was described with both an individual and family deductible, we required that both deductibles exceed the threshold for the plan to be classified as a HDHP. All other plans were considered low deductible health plans (LDHP). Such criteria are consistent with IRS regulations for minimum annual deductibles for health plans with paired tax-advantaged savings accounts during this time period and are similar to other thresholds used in industry reports (KFF, 2018). As we do not precisely observe the start and end of each plan’s benefit year and cannot link members in the same family, we are unable to analyze spending or utilization patterns before and after a deductible is fulfilled.

Among members in our sample, we analyze two cohorts. The first cohort (“stayers”) includes members who either had a low deductible during the entire study period or who had a high deductible during the entire study period. Hence, these groups had the indicated coverage for at least the study period, but a large fraction of them would be expected to have had such coverage for potentially much longer than the four years if the high rates of persistence in each coverage type we observe in the study period also prevailed before that period began. Hence, this cohort includes people with constant *levels *of cost sharing over potentially many more periods than other studies have been able to observe.

The second cohort (“switchers”) includes a smaller sample of members who were switched from a low to high deductible plan over the course of the study period. To address issues of plan selection, we also analyze a group of forced switchers from low to high deductible plans. These individuals had LDHP in earlier years and then had HDHP starting in 2016, 2017, or 2018 with no observed choice other than HDHP in the year of the switch. We identify forced switchers using employer group identifiers in our data, which allow us to observe which plan types (LDHP and/or HDHP) were available at each employer in each year. Forced switchers are individuals who: (1) had LDHP enrollment in earlier years, (2) switched to HDHP starting in 2016, 2017, or 2018, and (3) had no LDHP option available at their employer in the year of the switch, as observed by the absence of any LDHP enrollment among employees at that employer-year. We believe this variation in forced switching arises from employer decisions to discontinue LDHP options during our study period, while other employers maintained both plan types throughout.

Selective attrition could bias our comparison of spending *growth *between HDHP and LDHP stayers if individuals with different spending profiles have differential probabilities of remaining in the sample. Appendix Fig. 6 shows average spending across stayers, joiners, and leavers in each plan type. We observe that HDHP leavers tend to have lower spending than HDHP stayers, while LDHP leavers tend to have higher spending than LDHP stayers.

This pattern of selective attrition could attenuate estimated differences in spending *growth *between the two groups through a Roy model mechanism. Specifically, the retention of higher spenders in HDHPs and lower spenders in LDHPs would bias HDHP spending *growth *upward and LDHP spending *growth *downward, potentially masking a true HDHP effect on *growth*.

### Subgroup analysis

In addition to the primary definition of a high deductible described above, we also completed a secondary analysis of members in the cohort of “stayers” who were enrolled in plans with more extreme deductible *levels*. Specifically, we considered members exclusively enrolled in three types of plans: (1) those with “very high” deductibles, defined as an individual/family deductible ≥ $3,000/$6,000; (2) those with “very low” deductibles, defined as an individual/family deductible ≤ $250/$500; and (3) those with no (i.e., $0) deductibles. The first group represents a subgroup of the overall HDHP stayers group, the second group represents a subgroup of the overall LDHP stayers group, and the third group represents a subgroup of both (2) and the overall LDHP stayers group.

Within commercial insurance, the scope of care to which a deductible applies varies. Plans may have a single deductible that applies only to medical care, separate deductibles for medical and pharmacy care, or an integrated deductible that applies to both medical and pharmacy care. All three of these plan types exist at Elevance Health. In the plan benefit design data, we observe individual and/or family deductibles for each plan. We know with certainty that the deductibles apply to medical care; however, we cannot precisely identify whether the same deductibles also apply to pharmacy care. However, high deductible health plans offered in combination with a tax-advantaged health savings account (HSA) are required by law to apply the plan deductible to all services, including prescription drugs.^5^ As we are able to observe the presence of a paired HSA for some members in our overall HDHP group, we conducted an additional subgroup analysis of cost *growth *for such members.

## Estimation

### Sample

Our final study sample consists of 159,917 HDHP stayers, 164,574 LDHP stayers (63,214 without choice), and 12,888 switchers (4,653 forced switchers). Basic demographic characteristics are summarized in Table 1. Just under half of each cohort is female, with an average age of 42 years (stayers in HDHP and LDHP) and 44 years (forced switchers). While all cohorts include members across the country, a larger share of HDHP members reside in the Midwest region and a larger share of LDHP members reside in the West region. While the age distribution is similar in HDHP and LDHP, HDHP members are slightly healthier as judged by Charlson comorbidity scores (*p* < 0.001).

**Table 1:** Sample characteristics

	Descriptive analysis			Difference-in-differences analysis	
	HDHP	LDHP	Switchers	LDHP No choice	Forced switchers
*N*	159,917	164,574	12,888	63,214	4,653
Female	48.5%	49.8%	49.8%	49.7%	49.4%
Age					
Mean	42.33	42.13	43.48	43.47	43.98
SD	12.16	12.23	12.09	12.41	12.28
Age group					
18—24	8.7%	9.1%	9.5%	9.6%	10.5%
25—29	6.0%	5.8%	5.8%	6.3%	5.1%
30—34	9.3%	9.4%	10.0%	10.0%	8.5%
35—39	10.8%	11.0%	11.0%	11.4%	9.5%
40—44	11.7%	12.3%	12.3%	12.4%	12.0%
45—49	13.7%	13.8%	13.8%	13.4%	13.9%
50—54	15.3%	14.7%	15.3%	14.1%	16.7%
55—59	15.4%	14.3%	15.2%	13.4%	16.4%
60 plus	9.0%	9.5%	7.1%	9.5%	7.5%
Region					
Northeast	9.5%	7.4%	12.9%	10.5%	7.0%
Midwest	40.8%	22.2%	35.3%	24.4%	55.7%
South	23.5%	28.9%	23.6%	26.9%	21.6%
West	26.1%	41.4%	28.0%	38.2%	15.8%
Unknown	0.1%	0.0%	0.2%	0.0%	0.0%
Charlson Score					
0	77.6%	74.5%	77.8%	75.4%	75.2%
1	15.4%	17.2%	15.6%	16.8%	16.9%
2	4.5%	5.2%	4.0%	5.0%	4.7%
3 +	2.5%	3.1%	2.6%	2.8%	3.3%

This table reports the mean demographic characteristics of the members in our descriptive analysis (columns 1—3) and difference-in-differences (column 4 and 5) study samples. *SD* Standard deviation. “Charlson Score” refers to the member’s Charlson comorbidity index or score. *HDHP* High deductible health plans; *LDHP* Low deductible health plans. “Switchers” refer to all LDHP to HDHP switchers. “LDHP No Choice” refer to LDHP stayer members with no observed choice. “Forced Switchers” refer to LDHP to HDHP switchers with no observed choice

**Fig. 1:**
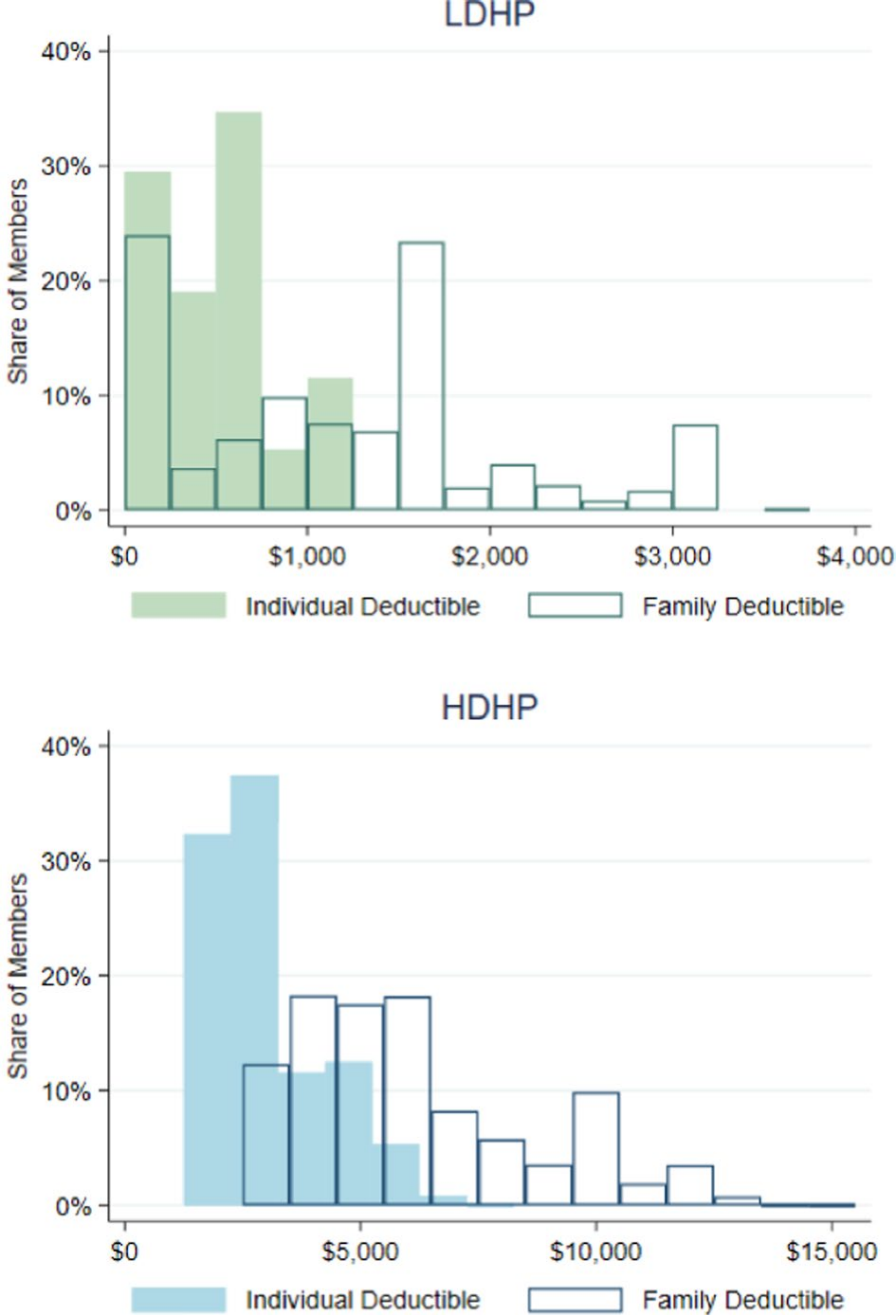
Average deductibles for LDHP and HDHP members. This figure shows the distribution of individual and family deductibles for members in our HDHP and LDHP stayer groups using an enrollment-weighted average for each member; HDHP = High deductible health plans, LDHP = Low deductible health plans

Figure 1 illustrates the distribution of individual and family deductibles for members in the HDHP and LDHP stayer groups. As members in each group may have multiple low or multiple high deductibles during the study period, this figure uses an enrollment-weighted average for each member. Approximately 20% of LDHP members have $0 deductibles and very few have individual deductibles over $1,000 (but below the $1,250 threshold). Deductibles in the HDHP group exhibit more variation across a wider range, with individual deductibles ranging from $1,250 to $7,350, and family deductibles ranging anywhere from $2,500 to $15,000. Median individual deductibles are $500 (LDHP) and $2,700 (HDHP), while corresponding median family deductibles are $1,150 and $5,700.

Consistent with the patterns in deductibles above, Table 2 shows that the average share of spending paid out-of-pocket is higher among members of the HDHP group relative to the LDHP group. For medical spending, the HDHP and LDHP out-of-pocket shares were 24% and 14% respectively in 2015, and for pharmacy spending they were 22% and 12%. The similar differences in out-of-pocket shares is consistent with most plans having integrated deductibles that apply to both medical and pharmacy spending. Increases in spending in either category over the four-year period were associated with increases in out-of-pocket payments however, the share of the increase paid out-of-pocket was relatively low, resulting in a 2—4 percentage point decline in out-of-pocket shares over the 4-year period in both groups. To the extent that the share of total expenses paid out-of-pocket is decreasing in total costs, this suggests that spending *growth *was associated with larger spending per episode of illness rather than more frequent spending of small amounts. This result is consistent with the hypothesis that spending *growth *was associated with more costly technologies as substitutes for less costly ones.

**Table 2:** Patient out-of-pocket shares by setting

	2015	2016	2017	2018
LDHP				
Medical	14.2%	13.6%	13.0%	12.2%
Pharmacy	11.7%	10.7%	10.8%	10.3%
Total	13.5%	12.8%	12.3%	11.7%
HDHP				
Medical	24.5%	22.6%	21.4%	20.5%
Pharmacy	21.7%	19.6%	18.3%	17.1%
Total	23.8%	21.8%	20.6%	19.6%

This table reports the average patient out-of-pocket share of spending in the settings of medical, pharmacy, and total for each year of the study period (2015—2018). “Total” includes both medical and pharmacy settings. *HDHP* High deductible health plans; *LDHP* Low deductible health plans

### Descriptive analysis

Our analysis begins with simple comparisons of trends and means for total, medical and pharmacy spending for HDHP and LDHP enrollees as a whole, and also for select subgroups, such as those for which choice is observed, those with very low or no deductible, those with very high deductibles, and those for whom an HSA is observed. The demographic characteristics for all of these subgroups are similar to their respective comparison group. Finally, we compare the means and medians for those enrollees who are observed to switch plans the year before and the year after they switch, and we also plot their spending trends. The results from these analyses are presented in Section "Results" . Concerns about selection limit our ability to make causal conclusions from descriptive analyses, and therefore, our strategy to address selection is described in the next subsection.

### Addressing plan selection

In any setting in which people are not randomly assigned to a particular insurance plan, the possibility of selection bias will frustrate attempts to make causal inferences from descriptives. Observed relationships between plan-level cost-sharing and total costs may be affected by choices of employers to attract and retain workers in competitive labor markets, choices of workers to accept jobs based on different benefits packages, or choices of workers to enroll in one of several plans offered by a given employer. If individuals are risk neutral but incremental premiums for plans with lower cost-sharing are not risk-rated, adverse selection is possible. In contrast, there may be favorable or advantageous selection if people who are more risk averse take precautions against risky behavior but attach high value to financial protection.

Our data from Elevance Health employer plans permits selection of some type to occur for some individuals in the sample. A possible outcome is adverse selection based on *levels *of health risk. Those who are at higher risk at the time when their employment and/or insurance is chosen will tend to choose plans with lower cost-sharing, all else equal. These individuals will then be observed to have higher spending *levels* relative to those lower-risk individuals who elected to enroll in plans with higher cost-sharing. However, if relative risks, expected benefits and insurance loading (difference between expected benefits and incremental premium) are constant over time, there need be no relationship between coverage chosen and *growth *in spending, assuming the expected benefits from new technology (the reason for spending *growth*) are distributed independently of risk. In other words, individuals of different risk *levels *should have the same *growth *in spending or benefits. Conversely, risk will be related to benefits *growth *to the extent that new technology is biased towards individuals with relatively higher or lower risk. For example, advances in preventive care will appeal to low risks, while new cancer treatments will appeal to high risks.

We explore the potential role of selection in our data by analyzing members within our overall LDHP and HDHP stayer groups for which we observe a choice of deductible level (high or low) at their employer. As we do not directly observe the plan menu at each employer, we infer annual choice of deductible based on observed simultaneous uptake of both high and low-deductible Elevance Health plans among employees of a given employer in the data. We do not classify members from firms for which we only observe fewer than 25 associated members, as the probability of observing take-up of multiple plans (if offered) is lower. Using this approach, we find that 53% of members across both plan types (HDHP and LDHP) never have a choice of deductible during the study period and 26% always have a choice of deductible. The remaining 21% of members have choice during some but not all years.

This same approach allows us to identify forced switchers for our main difference-in-differences analysis. Forced switchers are individuals who switched from LDHP to HDHP during the study period and had no observed LDHP option available at their employer in the year of switch. Specifically, we observe: (1) the individual's enrolled plan type and deductible level in each year from the claims data, and (2) whether other plan types were simultaneously available at their employer, based on the enrollment patterns of other employees at the same employer. When an individual switches to HDHP and we observe no LDHP enrollments among any employees at that employer in the switch year, we classify this as a forced switch.

This approach has two qualifications. First, it is possible that a firm offers multiple Elevance Health plans but not all plans are available to all employees (e.g. if certain business units or salary *levels *within the firm are offered different options than others). Second, it is possible that a firm offers insurance products from a non-Elevance Health insurer in addition to Elevance Health. In the first case, we may incorrectly infer that choice was present, while in the second case, we may incorrectly infer that choice was absent. While we expect the degree of misclassification resulting from these two cases to be minimal, we cannot rule it out entirely.

In addition to exploiting the presence or absence of choice, we further correct for selection bias on unobserved risk components with our analysis of the cohort of members who are forced to switch from low to high deductible plans. For this group, we observe the distribution of spending before and after the switch, which if constant over time, or shifts according to time-variant observables, allows us to estimate the change in spending due to switching plans with different *levels *of deductible, while also controlling for time-invariant unobservable characteristics. Exploiting the panel structure of our data and information about individuals who only have a HDHP plan option, we therefore use an event study difference-in-difference approach proposed by Callaway and Sant’Anna (2021) to properly account for the differential timing of treatment and heterogenous treatment effects over time. This addresses the biases of two-way fixed effect (TWFE) estimates when interventions are staggered (Goodman-Bacon, 2021; Callaway & Sant’Anna, 2021; Sun & Abraham, 2020; Athey & Imbens, 2021), which can be decomposed as the variance-weighted average treatment effect on the treated (VWATT) and some weights can be negative ( Borusyak, Jaravel, and Spiess 2024; de Chaisemartin & D’Haultfoeuille, 2020). It allows us to identify any differences in spending *levels *or *growth *that are attributable to plan type (HDHP or LDHP).

The Callaway and Sant'Anna (CS) approach differs from traditional event study models with two-way fixed effects in important ways. Rather than relying on a standard two-way fixed effects framework, the CS estimator constructs treatment effect estimates by comparing treated cohorts to appropriate control groups at each point in time, using doubly-robust estimation that combines regression adjustment with propensity score weighting. This approach controls for time-invariant differences between treatment and control groups through the comparison group design rather than through explicit unit fixed effects. We use bootstrapped standard errors in the CS estimator, which account for correlation within groups over time.

The estimator proposed by Callaway and Sant’Anna computes the group-time average treatment effect where a group is defined by the switchers who were forced into HDHP during the same time period (2016, 2017, or 2018 treatment cohorts). We aggregate these $ATT(g,t)$’s by time relative to the first year they switched to highlight treatment effect heterogeneity for an event study of spending *levels *and *growth*. Our control group is comprised of LDHP stayers without choice (“never-treated”). HDHP stayers and switchers currently in HDHP (“already treated”) are not included in the analysis. Results from switchers who have yet to be in HDHP (“not-yet-treated”) as a comparison group for those treated earlier are reported in the Appendix, and our overall results are robust to the type of control group used. This estimator is specifically designed for staggered adoption difference-in-differences settings and addresses concerns about treatment effect heterogeneity that arise in traditional two-way fixed effects models.

We use a non-parametric doubly robust difference-in-differences estimator. This approach requires a balanced panel and several assumptions. First, the treatment must be absorbing – once an individual is enrolled in a HDHP, that individual must remain in a HDHP. In our sample, we restrict to switchers who once they were limited to one observed plan option (HDHP), did not later have the choice to enroll in LDHP. Second, there should be limited anticipation of the HDHP treatment prior to the first year—we expect this to hold in our setting.^6^ Third, the treatment and control groups must have parallel trends conditional on covariates – we test for if pre-treatment group-time ATTs are statistically indistinguishable from zero. Finally, there must be common support, other shared characteristics, between the treatment and control groups – we report this in our summary statistics.

### Empirical model

To study healthcare spending and *growth*, we estimate the group-time average treatment effect ATT(g,t) which allows for treatment effect heterogeneity.

This is the average treatment effect for individuals belonging to a particular HDHP switcher group *g* at time period *t* and is expressed as follows (Callaway & Sant’Anna, 2021):

$$ATT(g,t) = E[{{Y}_{t}}^{1}-{{Y}_{t}}^{0}| {G}_{g}= 1]$$

Under certain assumptions described previously, using never treated individuals as the control group, and the doubly robust approach, it can be rewritten as:

$$\mathrm{A}\mathrm{T}\mathrm{T}(\mathrm{g},\mathrm{t};\updelta )=E\left[\left(\frac{{G}_{g}}{E{[G}_{g}]}-\frac{\frac{{p}_{g}(X)C}{1-{p}_{g}(X)}}{E\left[\frac{{p}_{g}(X)C}{1-{p}_{g}(X)}\right]}\right)\left({Y}_{t}-{Y}_{g-\updelta -1}- {m}_{g,t,\updelta }(X)\right)\right]$$

where

$${m}_{g,t,\updelta }(X) =E[{Y}_{t}-{Y}_{g-\updelta -1}| X,C= 1]$$

In the equation above, ${G}_{g}$ is a dummy variable equal to one if the individual is in group g of forced switchers to HDHP where $g$ is the year of first treatment or the individual’s HDHP switcher cohort date – 2016, 2017, or 2018. $C$ is a dummy equal to one if the individual is in the control group so they are not yet enrolled in HDHP or never enrolled in HDHP. By construction, either the individual is in the treatment group ${G}_{g}= 1, C = 0$ or in the control group ${G}_{g}=0, C = 1$.

We then take the weighted average of the ATT(g,t) to interpret the causal effect of HDHP. Our ATT(g,t)’s are aggregated by event time ($$e = t -g)$$ for an event study type of parameter that reports the average treatment effects by the length of exposure, *e*, for up to three years pre and post forced switch to a HDHP. This is the average effect of HDHP on spending *levels *for the group of switchers who were observed as having no choice in deductible for exactly *e* time periods. To draw conclusions about spending *growth*, we compare these effects at different time periods.

$${\theta}_{es}(e) = \sum_{g}^{T}\sum_{t}^{T}1\{g+e \le t\}P({G}_{g}= 1 | Treated for \ge e periods) ATT(g,t)$$

## Results

### Spending growth

#### Descriptive analysis

**Fig. 2:**
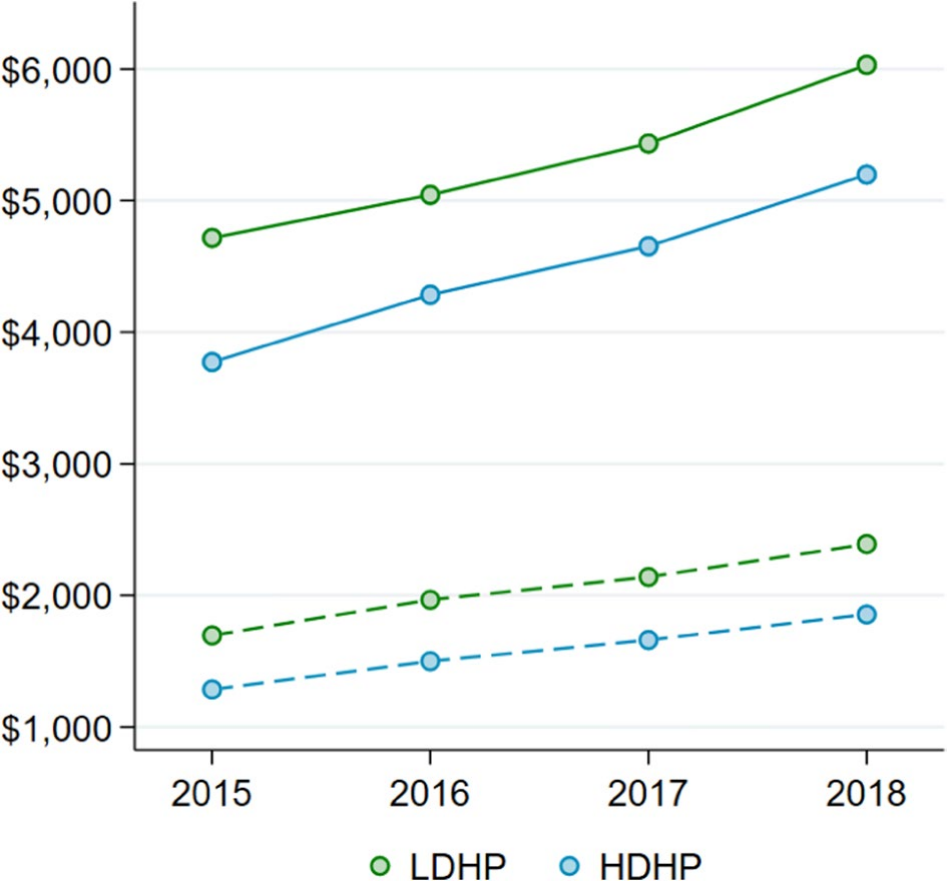
Trends in average annual spending per member. This figure shows the trends in average per-member medical and pharmacy spending for each year over the study period from 2015 to 2018; solid trends represent medical spending and dashed trends represent pharmacy spending. HDHP = High deductible health plans, LDHP = Low deductible health plans

**Fig. 3:**
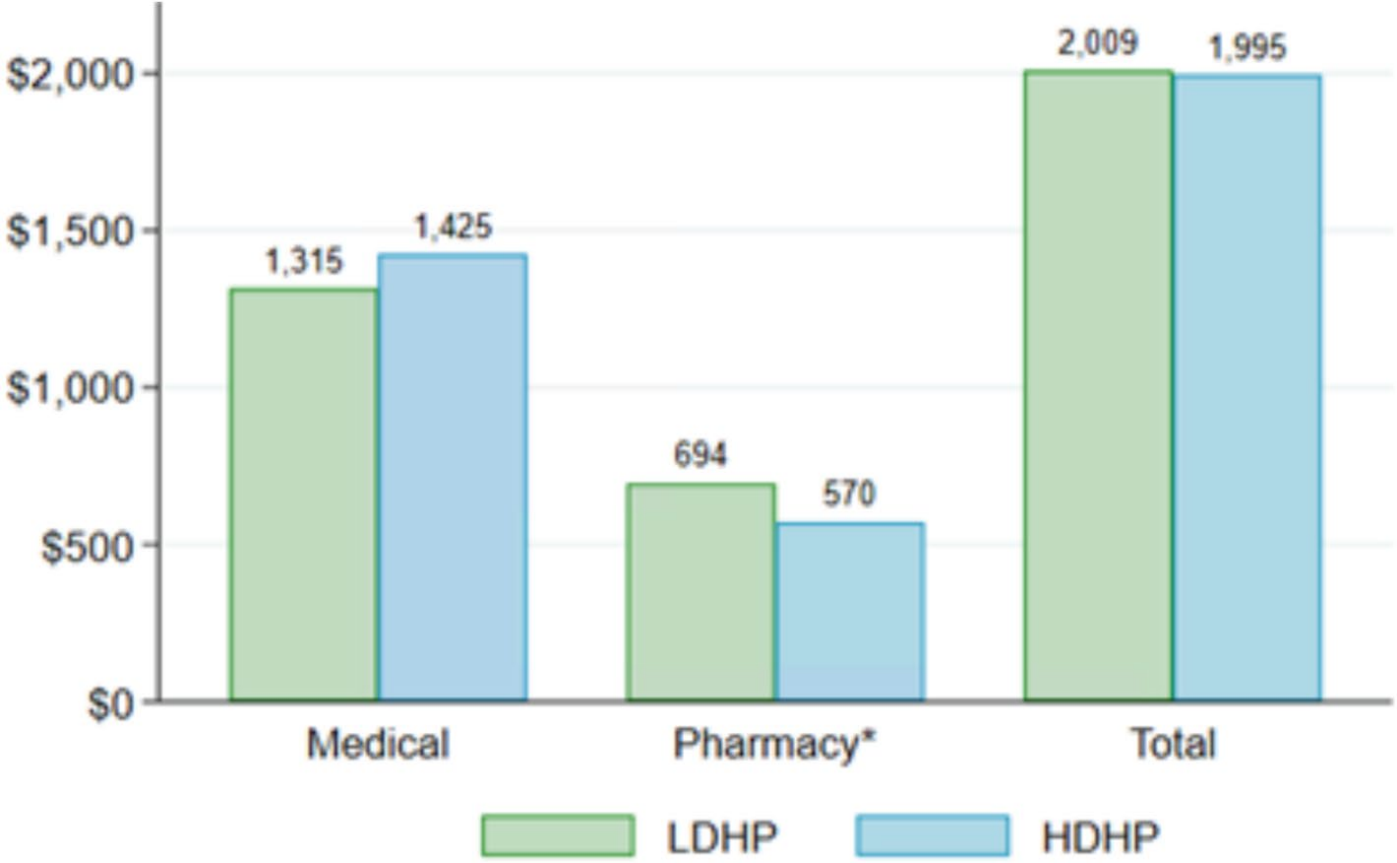
Average cost *growth *per member. This figure shows the average 3-year cost *growth *from 2015 to 2018 for members in our HDHP and LDHP stayer groups in absolute terms, unadjusted for any observable individual characteristics. “Total” includes both medical and pharmacy settings; * represents the difference in spending within the category is statistically significant with *p* < 0.01. HDHP = High deductible health plans, LDHP = Low deductible health plans

Figure 2 displays trends in average per-member medical and pharmacy spending for each year in the study period. Consistent with both adverse selection and moral hazard, both medical and pharmacy spending *levels *are approximately 20% lower in the HDHP group relative to the LDHP group. Both groups experienced *growth *in both types of spending over the study period as well. Figure 3 shows average 3-year cost *growth *in the two groups in absolute terms, unadjusted for any observable individual characteristics. Total spending *growth *is remarkably similar between the two groups, as judged by both measures. While *growth *in medical spending is also non-significantly different, pharmacy spending *growth *is significantly lower among HDHP members ($570 vs. $694 [3Y *growth*]; *p* < 0.01). It is worth noting that, especially within hospital-dominated total medical spending, episodes of care with new technology might more commonly have costs well above the deductible.

Tables 3 and 4 show spending *levels *and average *growth *for selected subgroups of our overall LDHP (Table 3) and HDHP (Table 4) groups. Relative to the overall group, average cost *levels *are higher among LDHP members with observed choice of deductible and lower among HDHP members with observed choice, consistent with some degree of adverse selection. Interestingly, members with “very low” and no deductibles have slightly *lower* spending *levels *but *higher* spending *growth* relative to the overall LDHP group. In contrast, members with “very high” deductibles have both lower spending *levels *and lower spending *growth*, relative to the overall HDHP group. HDHP members with HSAs, on the other hand, have higher baseline spending *levels *as well as higher *growth *in total spending. This higher *growth *results from higher *growth *in medical spending, and average *growth *in pharmacy spending is actually slightly lower relative to the overall HDHP group.

**Table 3:** LDHP subgroup analysis

	Overall LDHP	LDHP subgroups			
		Observed choice	No observed choice	Very low deductible	No deductible
*N*	164,574	42,628	63,214	51,614	36,897
Average Cost *Levels*					
Medical	$5,307	$5,370	$5,078	$5,305	$5,323
Pharmacy	$2,048	$2,047	$1,826	$1,991	$2,025
Total	$7,355	$7,417	$6,904	$7,296	$7,347
Average Annual *Growth*					
Medical	$438	$466	$472	$485	$524
Pharmacy	$231	$260	$212	$230	$236
Total	$670	$727	$685	$715	$760

This table reports spending *levels *and average *growth *for overall LDHP and LDHP subgroups. “Observed Choice” and "No Observed Choice" includes members of employers that respectively always and never offered HDHPs and LDHPs simultaneously each year of the study period (2015—2018); "Very Low" includes plans with individual/family deductibles ≤ 500; HDHP = High deductible health plans, *LDHP* Low deductible health plans

**Table 4:** HDHP subgroup analysis

	Overall HDHP	HDHP subgroups			
		Observed choice	No observed choice	Very high deductible	HSA
*N*	159,917	25,339	72,591	60,369	44,767
Average Cost *Levels*					
Medical	$4,478	$4,247	$4,278	$4,256	$5,121
Pharmacy	$1,575	$1,297	$1,492	$1,378	$1,596
Total	$6,053	$5,544	$5,770	$5,634	$6,717
Average Annual *Growth*					
Medical	$475	$494	$473	$475	$504
Pharmacy	$190	$179	$181	$170	$185
Total	$665	$674	$654	$645	$689

This table reports spending *levels *and average *growth *for overall HDHP and HDHP subgroups. “Observed Choice” and "No Observed Choice" includes members of employers that respectively always and never offered HDHPs and LDHPs simultaneously each year of the study period (2015—2018); *HAS* HDHP members with plan names explicitly indicating presence of a health savings account; "Very High" includes plans with individual/family deductibles ≥ 6,000; *HDHP* High deductible health plans; *LDHP* Low deductible health plans

**Fig. 4:**
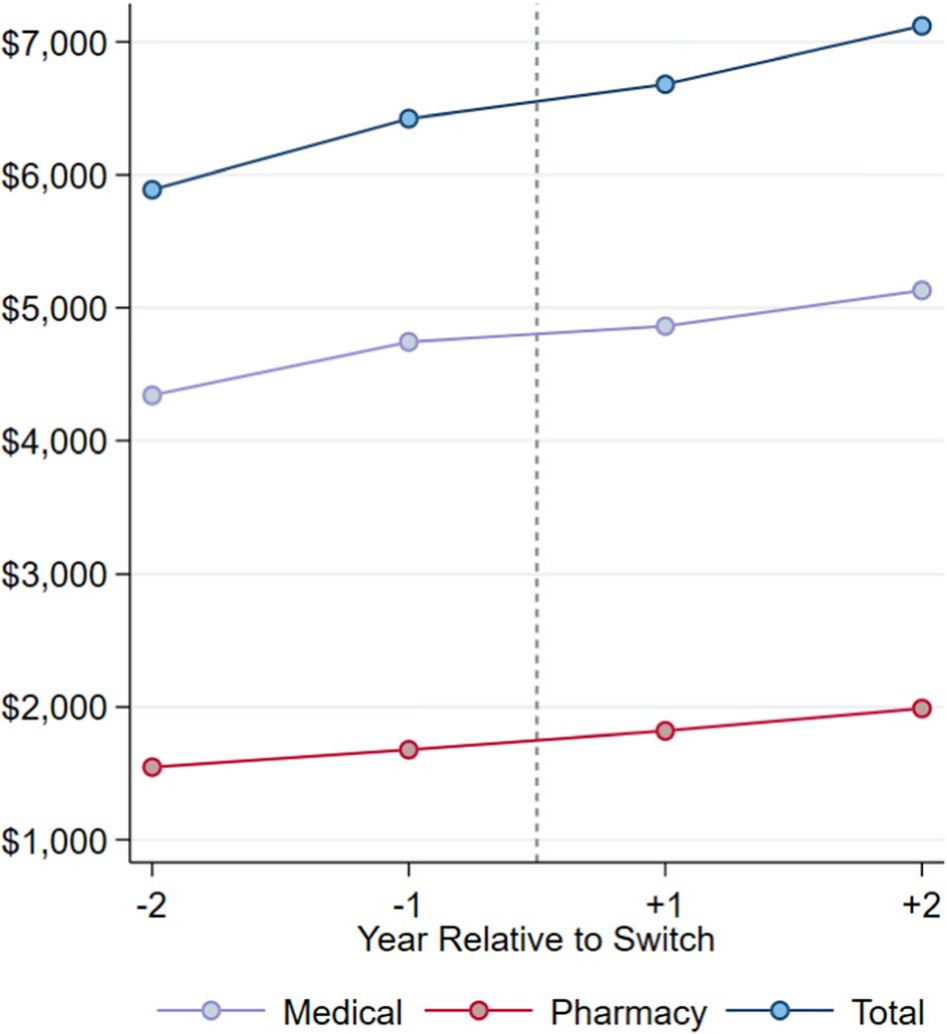
Average annual spending Pre- and Post-Switch from LDHP to HDHP. This figure presents trends in mean allowed costs per member including up to two years before and two years after the switch from a LDHP to a HDHP over the study period (2015—2018). “Total” includes both medical and pharmacy settings. HDHP = High deductible health plans, LDHP = Low deductible health plans

Table 5 shows summary statistics for the 12,888 members who switched from a LDHP to an HDHP over the study period. In the table, we compare mean and median spending *levels *in the year before switch to the year after switch. As expected, out-of-pocket spending increases considerably between the two years, with mean and median out-of-pocket spending per member increasing by 45% and 49% respectively. Conversely, both mean and median spending by Elevance Health (“plan” spending) decreased after the switch. Together, these effects led to a decrease of about 10% in median total allowed spending per member, but a slight increase in *mean* total spending per member. Figure 4 plots the trends in mean allowed costs per member including up to two years before and two years after the switch. The trends suggest that the decline in spending *growth *associated with switching from LDHP to HDHP is temporary, with spending *growth *returning to the pre-switch trend beyond 1 year.

**Table 5:** Mean and median annual spending per member pre- and post-switch

	Year before switch	Year after switch	Change	
			$	%
*A. Mean Costs*				
Medical				
Patient	$653	$929	$277	42%
Plan	$4,034	$3,894	-$140	−3%
Allowed	$4,743	$4,861	$118	2%
Pharmacy				
Patient	$182	$278	$96	52%
Plan	$1,494	$1,540	$46	3%
Allowed	$1,679	$1,821	$141	8%
Total				
Patient	$835	$1,207	$372	45%
Plan	$5,529	$5,434	-$95	−2%
Allowed	$6,423	$6,682	$259	4%
*B. Median Costs*				
Medical				
Patient	$191	$303	$111	58%
Plan	$577	$374	-$203	−35%
Allowed	$895	$836	-$59	−7%
Pharmacy				
Patient	$52	$63	$11	20%
Plan	$85	$34	-$51	−60%
Allowed	$177	$159	-$18	−10%
Total				
Patient	$357	$533	$176	49%
Plan	$1,024	$637	-$387	−38%
Allowed	$1,545	$1,393	-$152	−10%

This table reports mean and median annual spending *levels *for our “switchers” sample, members who switched from a LDHP to a HDHP during the study period (2015—2018), in the year before and the year after the switch. “Total” includes both medical and pharmacy settings. *HDHP* High deductible health plans; *LDHP* Low deductible health plans

### Dynamic difference-in-differences analysis

The descriptive results above show that, while spending *levels* differ between HDHP and LDHP, total spending *growth* does not. We further explore whether these results are robust to efforts to control for selection. We find in Fig. 4 that switching to a HDHP is associated with a one-time decrease (relative to LDHP) in *levels *of spending in year *t.* After this period, spending *growth *in the two types of plans is similar.

In Table 6 we report *ATT(g,t)* event-study estimates from members who were forced to switch from LDHP to HDHP as HDHP was their only observed choice (*N* = 4,653). Columns (1)-(3) show results for total, medical, and pharmacy spending, respectively, relative to the control group as never treated individuals with LDHP and no observed plan choice. For switchers who were not offered a choice of deductible, we observe a decrease in spending one year after switching for total and medical spending (marginally significant at *p* = 0.078 and *p* = 0.073, respectively). We find results consistent with the descriptive patterns in Fig. 4: a marginally significant decrease in spending for T + 1 for total and medical spending but no effect on subsequent spending *growth *(T + 2, T + 3). This empirical pattern—an immediate reduction in spending *levels *that does not persist, combined with no effect on subsequent spending *growth*—is consistent with our model's prediction. The model shows that expenditure on inputs with higher marginal health product exhibits lower coverage elasticity. Our findings therefore suggest that new technologies driving spending *growth *have higher marginal health product than the existing technologies that determine spending *levels*.

**Table 6:** Event study of dynamic treatment effects in aggregate spending

	Total	Medical	Pharmacy
T-2	843.8	992.3	−148.5
	(578.8)	(702.6)	(104.1)
T-1	379.8	470.1	−90.24
	(453.1)	(433.5)	(90.7)
T + 1	−775.8*	−773.9*	−1.899
	(429.6)	(447.5)	(71.0)
T + 2	125.7	116.1	9.611
	(465.8)	(458.4)	(124.0)
T + 3	−31.63	89.74	−121.4
	(555.8)	(459.1)	(181.0)
Control Group	Never Treated	Never Treated	Never Treated
*N*	271,468	271,468	271,468
Standard errors in parentheses	* *p* < 0.10	** *p* < 0.05	*** *p* < 0.01

This table reports the dynamic treatment effects in total, medical, and pharmacy spending from a difference-in-differences. The ATTs are estimated for each year relative to the “base-period” last pre-switch year with LDHP. The control group are “never treated” individuals at the same point in time. The time periods listed are relative to the year of switch from LDHP to HDHP with “T + 1” as the first year of HDHP. Our sample is comprised of individuals with LDHP stayer members with no observed choice (*N* = 63,214) and LDHP to HDHP switchers with no observed choice (*N* = 4,653)

We acknowledge that selective attrition could bias our comparison of spending *growth *between HDHP and LDHP stayers. As shown in Appendix Fig. 6, HDHP leavers have lower spending than HDHP stayers, while LDHP leavers have higher spending than LDHP stayers. This pattern could attenuate differences in spending *growth *between the two groups. However, our forced switcher analysis addresses this concern by following the same individuals before and after they switch plans, and finds the same pattern of results: a one-time reduction in spending *levels *with no effect on subsequent *growth*.

## Conclusion

Our results imply that the impact of high deductible health plans (compared to low deductible plans) on *growth *in spending over the period 2015—2018 was modest at most, with a one-time decrease in medical spending in the year following a switch to HDHP, and no effect on subsequent spending *growth*. These results suggest that the effect of switching to HDHPs is limited to a one-time reduction in spending on existing technology, with minimal impact on the adoption of new technologies driving subsequent spending *growth*. This finding is consistent with our model's prediction that expenditure on higher marginal product inputs exhibits lower coverage elasticity, and suggests that new medical technologies have higher marginal health product than established care. The implication is that, after a transition period in which spending *levels *are reduced, people in HDHPs experience the same *growth *in spending as those in more generous plans. There is some imprecision in our analysis, as in other work, regarding the exact length of the transition period, but no evidence supporting the hypothesis that HDHPs lower spending *growth *beyond that interval.

As with any observational study attempting to identify the impacts of non-randomly assigned interventions in a real-world setting, there are some limitations to these conclusions. Attempts to measure the anti-moral hazard effects of higher cost-sharing are always complicated by the possibility of self-selection into a high deductible plan by those with lower medical risks or propensity to seek care. While we attempt to account for such selection in multiple ways, we cannot disregard the possibility that some selection bias remains in our results. However, the likely direction of such bias is negative, and was not apparent in our results on spending *growth *for medical services or medical spending in the aggregate. Our measures of the impact of higher deductibles on the level of spending as people switch from low to high deductible plans are of the same order of magnitude as other estimates, suggesting little bias in the choice to switch or the timing of switch.

Our main analysis uses the Callaway-Sant'Anna (2021) estimator, which provides robust inference in our staggered adoption setting. While this approach controls for differences between treatment and control groups through its comparison group design, it does not fully substitute for employer fixed effects, which would control for time-invariant employer-specific characteristics that may influence plan offering decisions.^7^

More definitive welfare results than can be inferred from our results would require quantification of the cost-effectiveness of other new medical goods and services that are affected by coverage, along with measurement of the value of reduced risk protection (financial and health). In addition, the differences across plan type may well understate the impact of market wide shifts to higher or lower cost-sharing. Finally, while our aggregate analysis suggests HDHPs do not affect spending *growth *broadly, examining effects within specific patient populations affected by new diagnostic procedures and treatments remains an important area of future research.

## Figures and Tables

**Fig. 5:**
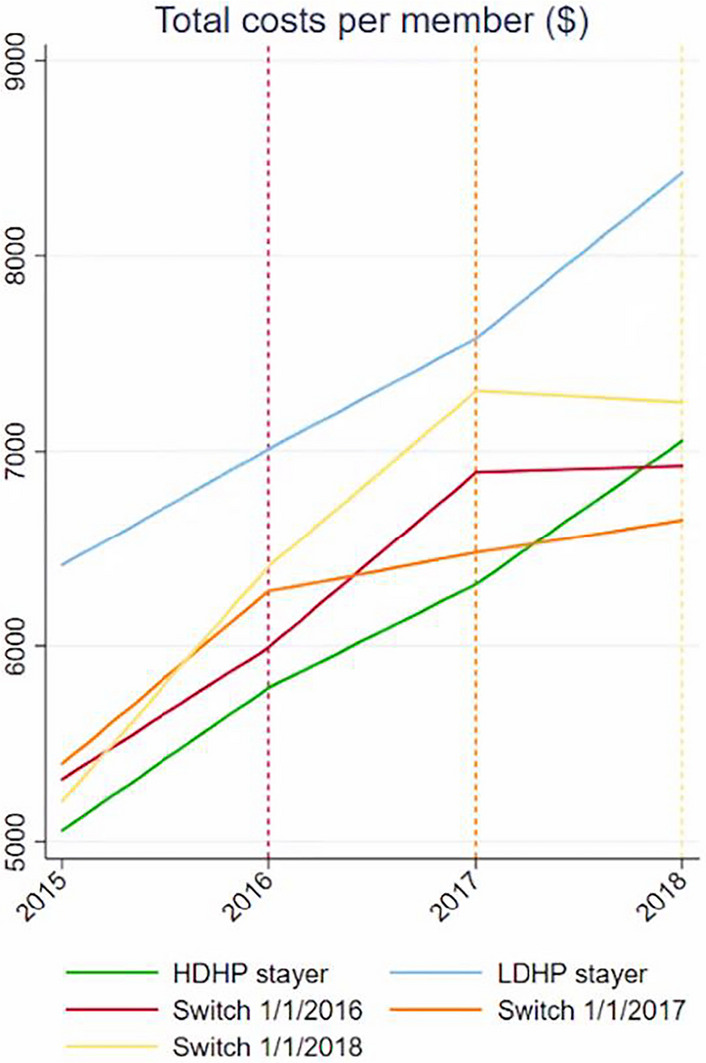
Average total costs per member by stayer and switcher groups. Notes: This table reports average total costs per member separately for HDHP and LDHP stayer and switcher groups by date of switch. HDHP = High deductible health plans, LDHP = Low deductible health plans. Switcher = switched from a low to high deductible plan over the course of the study period

**Fig. 6:**
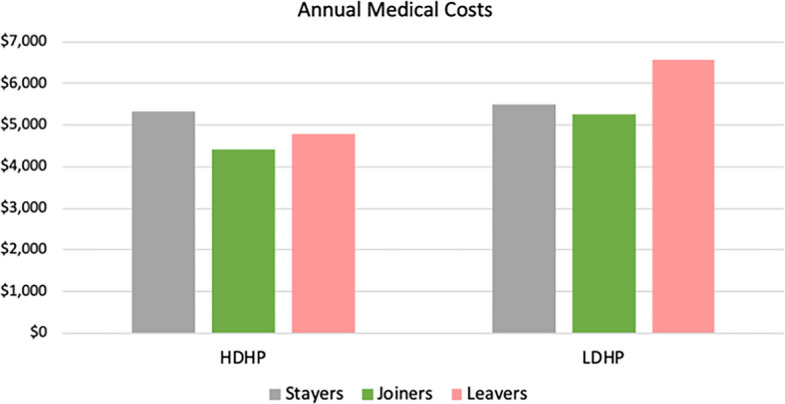
Average annual medical spending by plan groups. Notes: This table reports average values of spending separately for HDHP and LDHP stayer, joiner, and leaver groups. HDHP = High deductible health plans, LDHP = Low deductible health plans. Stayers are individuals who were continuously enrolled in HDHP (or LDHP) from 2015—2018. Joiners newly enrolled in a HDHP (or LDHP) for some period in 2015—2018. Leavers disenrolled in a HDHP (or LDHP) for some period in 2015—2018

## Appendix

**Table 7:** Linear regression models of spending

	No controls			With controls			With member fixed effects		
	Total	Medical	Pharmacy	Total	Medical	Pharmacy	Total	Medical	Pharmacy
	(1)	(2)	(3)	(4)	(5)	(6)	(7)	(8)	(9)
Year (vs. 2015)									
2016	636.2***	358.8***	277.4***	603.8***	334.0***	269.8***	605.0***	329.8***	275.2***
	(113.0)	(100.2)	(39.61)	(106.8)	(96.54)	(38.28)	(87.23)	(83.45)	(21.71)
2017	1310.1***	860.1***	450.0***	1254.9***	821.5***	433.5***	1277.7***	842.5***	435.2***
	(113.5)	(100.7)	(39.81)	(107.4)	(97.03)	(38.48)	(88.43)	(84.59)	(22.01)
2018	2141.7***	1468.6***	673.1***	2049.8***	1404.4***	645.4***	2051.2***	1391.6***	659.7***
	(113.6)	(100.8)	(39.85)	(107.5)	(97.14)	(38.51)	(88.89)	(85.03)	(22.13)
Deductible x Choice									
LDHP x Observed	576.4***	397.3***	179.1***	287.2*	185.3	102.0*	206.8	282.4*	-75.55*
	(123.8)	(109.8)	(43.40)	(117.1)	(105.8)	(41.97)	(141.8)	(135.7)	(35.30)
HDHP x Not Observed	1140.1***	-823.5***	-316.6***	-665.8***	-435.6***	-230.2***	105.0	82.89	22.06
	(113.1)	(100.3)	(39.66)	(107.8)	(97.39)	(38.62)	(139.7)	(133.7)	(34.78)
HDHP x Observed	1348.2***	-853.7***	-494.6***	-712.7***	-410.7***	302.0***			
	(132.2)	(117.2)	(46.36)	(125.1)	(113.1)	(44.84)			
Deductible x Choice x Year									
LDHP x Observed × 2016	-36.37	-16.96	-19.41	31.08	33.89	-2.817	33.57	31.06	2.511
	(172.1)	(152.6)	(60.33)	(162.7)	(147.1)	(58.31)	(134.8)	(129.0)	(33.56)
LDHP x Observed × 2017	-181.2	-180.4	-0.859	-59.35	-95.51	36.15	-103.8	-167.3	63.55
	(173.2)	(153.6)	(60.74)	(163.8)	(148.1)	(58.71)	(138.7)	(132.6)	(34.52)
LDHP x Observed × 2018	-108.9	-162.3	53.39	92.79	-22.89	115.7	106.4	-12.47	118.9***
	(175.1)	(155.3)	(61.39)	(165.6)	(149.6)	(59.33)	(140.8)	(134.7)	(35.05)
HDHP x Not Observed × 2016	-20.86	84.06	-104.9	51.95	141.0	-89.07	55.98	129.0	-73.00*
	(158.3)	(140.4)	(55.50)	(149.7)	(135.3)	(53.65)	(122.2)	(116.9)	(30.41)
HDHP x Not Observed × 2017	-164.2	-30.80	-133.4*	-77.70	33.38	-111.1*	-101.4	-7.045	-94.37**
	(157.5)	(139.7)	(55.24)	(149.0)	(134.7)	(53.39)	(122.5)	(117.2)	(30.49)
HDHP x Not Observed × 2018	-177.6	-36.87	-140.7*	-80.27	33.58	-113.9*	-99.07	12.78	-111.9***
	(156.8)	(139.1)	(54.98)	(148.3)	(134.0)	(53.14)	(122.6)	(117.2)	(30.51)
HDHP x Observed × 2016	193.7	204.1	-10.49	160.2	167.9	-7.782	142.9	195.0	-52.09
	(189.6)	(168.2)	(66.49)	(179.4)	(162.1)	(64.29)	(147.5)	(141.1)	(36.71)
HDHP x Observed × 2017	-90.26	-35.35	-54.91	-60.03	-36.43	-23.60	-105.2	-47.86	-57.36
	(194.8)	(172.8)	(68.32)	(184.3)	(166.6)	(66.07)	(153.9)	(147.2)	(38.31)
HDHP x Observed × 2018	-324.2	-143.1	-181.1**	-162.6	-59.35	-103.3	-151.0	-8.073	-143.0***
	(199.8)	(177.2)	(70.06)	(189.1)	(170.9)	(67.77)	(159.2)	(152.3)	(39.63)
Year Relative to Switch x Stayer/Switcher x Choice									
Year t—3 × Switch x Not Observed	-1231.7	-910.6	-321.2	-753.2	-600.7	-152.5	855.8	448.4	407.4*
	(632.7)	(561.1)	(221.8)	(599.3)	(541.7)	(214.8)	(801.6)	(766.8)	(199.5)
Year t—3 × Switch x Observed	-997.1*	-717.1	-280.0	-449.0	-300.0	-149.0	658.8	252.3	406.5*
	(499.7)	(443.2)	(175.2)	(472.6)	(427.1)	(169.4)	(720.9)	(689.6)	(179.4)
Year t—2 × Switch x Not Observed	-719.6	-279.0	-440.6**	-250.0	25.90	-275.9	1571.6*	1344.0*	227.6
	(463.7)	(411.2)	(162.6)	(439.0)	(396.8)	(157.3)	(701.1)	(670.7)	(174.5)
Year t—2 × Switch x Observed	-849.4*	-644.2*	-205.1	-150.0	-128.2	-21.86	1057.4	783.5	273.9
	(341.9)	(303.2)	(119.9)	(323.3)	(292.3)	(115.9)	(634.4)	(606.9)	(157.9)
Year t—1 × Switch x Not Observed	-869.0*	-470.0	-399.0**	-151.9	31.64	-183.5	1347.9*	1118.4	229.5
	(437.7)	(388.2)	(153.5)	(414.2)	(374.4)	(148.5)	(670.7)	(641.6)	(166.9)
Year t—1 × Switch x Observed	-769.6**	-534.4*	-235.3*	-119.3	-59.22	-60.07	1155.1*	944.6	210.5
	(264.9)	(235.0)	(92.90)	(250.6)	(226.5)	(89.83)	(581.5)	(556.3)	(144.7)
Year t + 1 × Switch x Not Observed	-211.9	-471.2	259.4*	-42.66	-307.6	264.9*	732.7	590.3	142.4
	(332.6)	(295.0)	(116.6)	(314.9)	(284.6)	(112.9)	(642.9)	(615.0)	(160.0)
Year t + 1 × Switch x Observed†	1804.4***	-986.2***	-818.3***	-797.5**	-292.3	-505.2***	954.5	858.6	95.91
	(307.2)	(272.4)	(107.7)	(290.5)	(262.6)	(104.1)	(571.3)	(546.6)	(142.2)
Year t + 2 × Switch x Not Observed	-425.1	-622.0	196.9	-30.83	-307.2	276.4*	1139.7	1031.5	108.2
	(366.7)	(325.2)	(128.6)	(346.9)	(313.6)	(124.3)	(654.7)	(626.3)	(163.0)
Year t + 2 × Switch x Observed†	2261.1***	1391.4***	-869.7***	-1206.5**	-662.9	-543.6***	257.2	303.0	-45.75
	(418.6)	(371.2)	(146.8)	(395.8)	(357.8)	(141.9)	(592.9)	(567.1)	(147.6)
Year t + 3 × Switch x Not Observed	-518.3	-636.2	117.9	-142.2	-327.9	185.7	622.6	643.9	-21.25
	(522.2)	(463.1)	(183.1)	(493.8)	(446.3)	(177.0)	(736.5)	(704.5)	(183.3)
Year t + 3 × Switch x Observed†	-2611.0***	-1755.2**	-855.8***	-1490.9**	-964.9	-526.0*			
	(606.2)	(537.6)	(212.6)	(573.1)	(518.0)	(205.4)			
Controls									
Sex	No	No	No	Yes	Yes	Yes	No	No	No
Age	No	No	No	Yes	Yes	Yes	No	No	No
Region	No	No	No	Yes	Yes	Yes	No	No	No
Charlson Score	No	No	No	Yes	Yes	Yes	No	No	No
Member Fixed Effects	No	No	No	No	No	No	Yes	Yes	Yes
Observations	1,070,428	1,070,428	1,070,428	1,069,644	1,069,644	1,069,644	1,070,428	1,070,428	1,070,428
Adjusted R-squared	0.002	0.001	0.002	0.108	0.073	0.067	-0.330	-0.332	-0.328
*P*-value for H0: β† = β† = β†	0.411	0.373	0.956	0.470	0.435	0.975			

This table reports results from our traditional event study specifications which includes members exclusively enrolled in a HDHP or LDHP (“stayers”) and members who switched from HDHP to LDHP (“switchers”). These supplementary models do not include employer fixed effects. Columns (1)-(3) do not control for observable and unobservable risk factors, Columns (4)-(6) include observable risk factors, and Columns (7)-(9) include member fixed effects. Standard errors are shown in parentheses; * *p* < 0.05, ** *p* < 0.01, *** *p* < 0.001. Coefficient estimates for controls are not shown. “Observed” and "Not Observed" includes members of employers that respectively always and never offered HDHPs and LDHPs. “Charlson Score” refers to the member’s Charlson comorbidity index or score. *HDHP* High deductible health plans; *LDHP* Low deductible health plans

**Table 8:** Baseline spending and *growth *for top drugs

	2015 *levels*				3Y *growth*			
	LDHP	HDHP	Difference	*p*-value	LDHP	HDHP	Difference	*p*-value
All Prescription Drugs	$1,696	$1,285	-$411	< 0.001	$694	$570	-$124	< 0.001
Top 100 with CE Study								
All (*N* = 86)	$786	$644	-$142	< 0.001	$348	$300	-$48	0.020
High-value (*N* = 57)	$560	$457	-$104	< 0.001	$214	$201	-$13	0.429
Low-value (*N* = 29)	$226	$188	-$38	< 0.001	$134	$100	-$35	0.007
Top 100 with CE Study + Positive *Growth*								
All (*N* = 57)	$468	$392	-$76	< 0.001	$526	$442	-$84	< 0.001
High-value (*N* = 41)	$370	$310	-$60	< 0.001	$340	$301	-$39	0.005
Low-value (*N* = 16)	$98	$82	-$16	0.015	$186	$141	-$45	< 0.001

This table reports average values of spending and change in spending for each category of drugs separately for our HDHP and LDHP stayer samples. “All Prescription Drugs” refers to all prescription pharmaceuticals. “Top 100 Drugs” are the top 100 drugs with highest total spending over the study period. “CE Study” are the drugs we classified into high- or low-value using the Tufts CEA Registry. “Positive Growth” refers to the drugs with positive spending growth from 2015—2018. High- and low-value drugs include those with an incremental cost-effectiveness ratio (ICER) below and above $50,000, respectively. The p-values reported in the last columns are from two-tailed t-tests of the difference in means for the LDHP and HDHP stayer groups, assuming equal variance. 3Y = Three-year change in total spending per member (2015—2018). *CE* Cost-effective. *HDHP* High deductible health plans; *LDHP* Low deductible health plans

**Table 9:** Two-part models of allowed costs—total and by setting (including member fixed effects)

	Total		Medical		Pharmacy	
	LPM	OLS	LPM	OLS	LPM	OLS
	(1)	(2)	(3)	(4)	(5)	(6)
Deductible x Choice						
LDHP x Observed Choice	0.01***	199.5	0.01***	288.3	0.02***	-106.4*
	(0.002)	(162.0)	(0.002)	(161.8)	(0.003)	(47.4)
HDHP x No Observed Choice	0.00	118.1	0.00	115.0	0.00	19.5
	(0.002)	(162.5)	(0.002)	(163.4)	(0.002)	(48.5)
Year (vs. 2015)						
2016	0.01***	636.8***	0.01***	319.6**	0.01***	378.6***
	(0.001)	(101.3)	(0.001)	(101.2)	(0.002)	(29.7)
2017	0.01***	1337.2***	0.01***	870.5***	0.01***	579.9***
	(0.001)	(102.3)	(0.001)	(102.2)	(0.002)	(30.0)
2018	0.02***	2203.5***	0.02***	1484.0***	0.02***	886.5***
	(0.001)	(102.3)	(0.001)	(102.2)	(0.002)	(30.0)
Year x Deductible						
2015 × HDHP	0.00	102.7	0.00	-22.33	0.00	136.2**
	(0.002)	(141.6)	(0.002)	(141.8)	(0.002)	(41.7)
2016 × HDHP	0.00	171.0	0.00	135.7	0.01**	28.4
	(0.002)	(139.6)	(0.002)	(139.6)	(0.002)	(41.0)
2017 × HDHP	0.00	30.85	0.00	-9.024	0.00	27.2
	(0.002)	(137.1)	(0.002)	(137.0)	(0.002)	(40.2)
Year x Deductible x Choice						
2016 × LDHP x Observed Choice	0.00	9.3	0.00	9.9	0.00	-27.2
	(0.002)	(158.2)	(0.002)	(158.0)	(0.003)	(46.2)
2016 × HDHP x Observed Choice	0.00	80.6	0.00	70.4	0.00	16.2
	(0.002)	(173.1)	(0.002)	(174.1)	(0.003)	(51.5)
2017 × LDHP x Observed Choice	0.00	-142.2	0.00	-241.9	0.00	46.8
	(0.002)	(161.1)	(0.002)	(160.8)	(0.003)	(47.1)
2017 × HDHP x Observed Choice	0.00	-69.6	0.00	-117.3	0.01**	34.5
	(0.002)	(180.3)	(0.002)	(181.2)	(0.003)	(53.8)
2018 × LDHP x Observed Choice	0.00	139.7	0.00	0.8	0.00	126.6**
	(0.002)	(162.4)	(0.002)	(162.2)	(0.003)	(47.5)
2018 × HDHP x Observed Choice	0.00	-61.6	0.00	-59.06	0.01***	-44.2
	(0.002)	(186.4)	(0.003)	(187.5)	(0.003)	(55.7)
N_i_	257,221	250,896	257,221	248,427	257,221	239,842
N_it_	1,028,884	913,831	1,028,884	879,126	1,028,884	798,361

*LPM* Linear probability model; *OLS* Ordinary least squares; standard errors in parentheses; **p* < 0.05, ***p* < 0.01, ****p* < 0.001

## Footnotes

1 We assume perfect agency or a process of joint decision making between patients and physicians, where clinical and financial aspects are discussed in the choice of treatment.

2 We use the parameter $\upalpha$ as a summary measure of plan generosity to avoid (unnecessary) complexities of the nonlinearities introduced by deductibles.

3 Evidence has shown that physician practice style, as a proxy for advice, is often uniform across patients regardless of their particular insurance coverage or other demand parameters. If physician advice is tailored to the average or typical patient’s insurance coverage, rather than to that of each individual patient, an individual patient’s deductible may have an attenuated effect on spending relative to that of other patients with different deductibles—compared to the effect of changes in cost sharing in the market as a whole. Finkelstein (2007) demonstrated this kind of “norms” behavior exists and leads to impacts of market-level changes in insurance coverage on spending larger than what would be implied by studies of individual responses to cost-sharing. Hence, any impacts we detect that arise from variations in individual insurance coverage can be interpreted as a lower bound to the potential impacts of market-level changes.

4 A minority of members had missing benefit design data at the beginning, middle or end of the study period. For such members, we imputed benefits based on benefits observed closest to the missing time period.

5 Exceptions are permitted for preventive medications.

6 Employees usually learn about benefit design changes during limited Open Enrollment periods in the Fall. We do not have specific information about employees’ knowledge about the switch or characteristics about the employers. We include analysis using alternative switcher groups and descriptive trends in spending by switcher groups in the Appendix.

7 Our supplementary analyses in Appendix Table 7 present traditional event study specifications with member fixed effects but similarly do not include employer-level fixed effects.
